# The Treatment of Trachoma

**Published:** 1908-08-29

**Authors:** 


### Ophthalmology.

### THE TREATMENT OF TRACHOMA.

By some trachoma is considered incurable. This may be true of that stage where obstinate sequelæ are well marked, but of the earlier stages it is inaccurate. The cure of trachoma requires the earliest possible treatment of the disease; that is, by removal of the trachomatous infiltration before scarring and pannus have arisen. At the same time good hygienic surroundings and general measures are of great importance. Treatment may extend over several years before a cure is accomplished: much depends upon the regularity and intelligence with which it is carried out. For the most part the sufferers belong to the lower classes and have neither patience, time, nor means for sufficiently long treatment. They undergo treatment for a short time, the condition improves, and then they leave off; a relapse occurs, they submit to treatment for a time, but they never become really cured.

The treatment must be adapted to the stage and special form of the disease. The rapid cure of any discharge must be attempted for prophylactic reasons. Whatever method is adopted, the whole of the diseased area must be attacked. The "mild" cases, in which there are no inflammatory symptoms, no follicles in the tarsal conjunctivæ, only a few in the retrotarsal folds, and no secretion, respond most readily to treatment. In this early stage, when the follicles are hard and unripe, they cannot well be expressed without considerable injury to the conjunctiva. Destruction of the conjunctiva is to be avoided and resorption of the trachoma bodies is to be aimed at. This is best obtained by an application of the copper stick, a smooth cone of copper sulphate fixed in a handle. It is to be used once a day (a drop of cocaine, 2 per cent. may first be instilled), and a mild lotion of boracic acid can be used if necessary. It is to be used with care; indiscriminate use for too long a period is to be avoided, since too strong a reaction ensues and retards a cure. The application should be made lightly and equally over the conjunctiva, taking care to go well into the fornices and to avoid touching the cornea. The use of the copper stick is continued, unless the reaction is too strong, until the follicles disappear. If the reaction is greater than is desired, the copper may be replaced by the alum stick. When all follicles have disappeared treatment should be stopped for a time, and resumed at once if there is any recurrence.

The use of $x$-rays in some of these early cases is very beneficial. The patient is seated, with the face about 9 inches from the tube. The lids may be everted or the application may be made through the lids. The latter method is more comfortable to the patient, and the effect is equal to that obtained by the exposed method. The sitting is of 5 minutes' duration, and is continued daily for a week, unless there is a marked reaction before then. The following week no treatment is given, except perhaps boracic acid lotion. Several cases have been cured in this way, in others again the best results were got by combining the $x$-ray and copper stick methods, a better response being obtained to the copper after the $x$-rays. Occasionally the $x$-rays produce an exacerbation of the disease, but on the whole they are of no little value. The same cannot be said of the use of radium and high frequency current.

In cases where the follicles are more marked and ripe, expression should always be done at the outset. The procedure is simple and can be done under cocaine, applied superficially or by submucous injection. Grady's expression forceps is perhaps the most useful. In expression the epithelium over the follicle is ruptured and the contents expelled. When this method is employed correctly the removal of the folicles and diffuse infiltration is not followed by excessive scarring. After expression the conjunctiva is painted with a 1 per cent. solution of hydrarg. perchlor. in glycerin. This is followed by considerable reaction for a few days, and produces a discharge, which is to be treated with boracic acid lotion. Afterwards copper and alum are to be used as described above. Expression can be usefully repeated, as the follicles gain mastery over the copper; they are to be looked for in the retrotarsal folds, especially at the inner and outer angles.

Mechanical friction, such as massage of the conjunctiva with dry boracic acid powder, is not of much value; nor is scarification to be recommended, on account of the injury inflicted on the conjunctiva.

Expression gives no guarantee against re-infection, and after-treatment with medicaments is necessary, as after other methods. The predilection of trachoma for the fornices was early noted, and it is there that exacerbations and recurrences take their origin and proliferation of the conjunctiva is observed. When this is marked, excision of the fornices has proved of considerable value; care must be taken not to remove any of the bulbar conjunctiva. Other sequelæ, as trichiasis, entropion, blepharophimosis, blepharitis, or implications of the lachrymal passages, require appropriate treatment by operation or otherwise.

More severe cases, where inflammatory symptoms are present, such as marked swelling and injection of the conjunctiva, with more or less profuse purulent or muco-purulent discharge, are first to be treated as a muco-purulent conjunctivitis. Antiseptic lotions (hydrarg-perchlor. 1 in 6,000) frequently, 2 per cent. silver nitrate once a day, and a simple ointment to prevent the lids sticking together, are to be used. When these acute symptoms have subsided, the combined copper and expression treatment is begun. In advanced gelatinous trachoma, and in cases where there is scarring, with healed conjunctiva, but a thickened and much infiltrated tarsus, Kuhnt's method of excising the tarsus is very valuable.

When the limbus is much swollen, and the edge of the cornea vascularised and infiltrated, corneal complications may ensue. Irritating treatment should then be stopped, and atropine used to lessen the tendency to iritis. In obstinate pannus, peritomy, with scissors or galvano-cautery, is useful, or the acute inflammatory reaction produced by the instillation of jequiritol into the conjunctival sac.

